# Complete Genome Sequence of *Rhodococcus* sp. Strain IcdP1 Shows Diverse Catabolic Potential

**DOI:** 10.1128/genomeA.00711-15

**Published:** 2015-07-02

**Authors:** Jie Qu, Li-Li Miao, Ying Liu, Zhi-Pei Liu

**Affiliations:** a State Key Laboratory of Microbial Resources, Institute of Microbiology, Chinese Academy of Sciences, Beijing, China; b University of Chinese Academy of Sciences, Beijing, China

## Abstract

The complete genome sequence of *Rhodococcus* sp. strain IcdP1 is presented here. This organism was shown to degrade a broad range of high-molecular-weight polycyclic aromatic hydrocarbons and organochlorine pesticides. The sequence data can be used to predict genes for xenobiotic biodegradation and metabolism.

## GENOME ANNOUNCEMENT

*Rhodococcus* is a metabolically versatile genus with remarkable ability to catabolize a wide range of organic compounds (1). Several *Rhodococcus* strains have been isolated from various contaminated environments (2–6). *Rhodococcus* sp. strain IcdP1 was isolated from contaminated soil of the abandoned Beijing Coking Plant in China. It can degrade numerous high-molecular-weight polycyclic aromatic hydrocarbons (HMW-PAHs), such as benzo[*a*]pyrene and indeno[1,2,3-*cd*]pyrene, and organochlorine pesticides (OCPs), including hexachlorocyclohexanes and 1,1,1-trichloro-2,2-bis(4-chlorophenyl) ethane. It is useful in the bioremediation of contaminated environments.

Whole-genome shotgun (WGS) DNA sequencing was performed by Majorbio (Shanghai, China) using paired-end sequencing with HiSeq 2000 (Illumina) and long sequencing with PacBio RS (Pacific Biosciences). Three Illumina libraries, two paired-end libraries (300 bp and 800 bp) and a 2-kb mate pair library were constructed and sequenced, giving approximately 219-fold genome coverage (BioProject numbers SRX997125, SRX1009942, and SRX1009943, respectively). An approximately 8- to 10-kb-insert PacBio library was constructed and sequenced on an 8 single-molecule real-time (SMRT) cell, yielding 119,255 reads (average length, 7,446 bp) and >150× genome coverage (BioProject number SRX1009944). *De novo* assembly of the Illumina and PacBio reads was carried out using a novel correction algorithm and assembly strategy (7). The Illumina sequences were assembled into 235 contigs, with an *N*_50_ length of 74,570 bp by SOAP*denovo* version 2.04 (8), and the contigs were joined into 18 scaffolds. The error inherent in long single-molecule sequences was corrected using BLASR, and then the PacBio-corrected reads (PBcR) were *de novo* assembled into scaffolds alone by overlapping sequencing reads and subsequently assembled with Celera Assembler 8.0 (7).

The genome was analyzed by Rapid Annotations using Subsystems Technology (RAST) (9). The genome size was 5,922,928 bp, comprising 5,480 open reading frames and 430 subsystems. The G+C content was 70.62%. Fifty-four tRNAs were identified by tRNAscan-SE version 1.3.1 (10), and 12 rRNAs were predicted by Barrnap 0.4.2. RAST annotation indicates that *Rhodococcus opacus* B4 (score, 515) and *Rhodococcus jostii* RHA1 (score, 485) are its closest neighbors. The subsystem categories representing the metabolisms of carbohydrates; amino acids and derivatives; and cofactors, vitamins, prosthetic groups, and pigments are the most abundant, as they account for 631, 622, and 451 proteins, respectively. Three hundred eighty-eight open reading frames (ORFs) are involved in the metabolism of fatty acids, lipids, and isoprenoids, while 145 ORFs are involved in the metabolism of aromatic compounds. In addition, the genome contains at least 50 dioxygenase genes and 20 putative cytochrome P450 genes, which enables it to initiate the oxidation of persistent organic pollutants. Analysis of the genome of IcdP1 will help us to identify diverse biodegradation genes and, further, to elucidate the degradation pathways for HMW-PAHs and OCPs.

### Nucleotide sequence accession number. 

The complete genome sequence of *Rhodococcus* sp. strain IcdP1 has been assigned GenBank accession no. CP011341.
